# Regulatory NLRs in HSV-1 Infection: Direct Evidence, Comparative Mechanisms and Testable Hypotheses

**DOI:** 10.3390/pathogens15090908

**Published:** 2026-08-28

**Authors:** Mengting Zhu, Shuai Zhao, Xing Sun, Shuhan Wang, Shiyuan Hou, Xing Shen, Jielin Zhou, Ziyu Liu, Xingan Wu, Rongrong Liu

**Affiliations:** 1 Department of Microbiology, School of Basic Medical Sciences, Fourth Military Medical University, Xi’an 710032, China; zhumengting1117@163.com (M.Z.); zhaoshuai202510@163.com (S.Z.); hsyw0319@163.com (S.H.); shenxing11111@163.com (X.S.); m15596682921@163.com (J.Z.); 2 School of Medicine, Northwest University, Xi’an 710032, China; 3 School of Basic Medical Sciences, Fourth Military Medical University, Xi’an 710032, China; sunxing0410@163.com (X.S.); 19567685692@163.com (S.W.)

**Keywords:** herpes simplex virus, herpesvirus, regulatory NLR, NLRC3, NLRC5, NLRX1, cGAS-STING, MHC class I, MAVS, innate immunity

## Abstract

Herpesviruses establish lifelong persistence through acute replication, immune evasion, latency, and reactivation. Nucleotide-binding oligomerization domain-like receptors (NLRs) are most commonly discussed in the context of inflammasome sensors, pyroptosis, and IL-1 family cytokines. Here, we depart from this canonical framework to focus on three regulatory NLRs—NLRC3, NLRC5, and NLRX1—that act outside inflammasome assembly. Using HSV-1 as the central model and other herpesviruses as comparators, we propose a conceptual framework in which these NLRs modulate three host-control layers: cGAS-STING-dependent DNA sensing, MHC class I antigen presentation, and MAVS-mediated mitochondrial antiviral signaling. We explicitly frame these NLRs as regulatory hypotheses for HSV-1 biology rather than established restriction factors, and we provide prioritized, falsifiable predictions to guide future experimentation. Current evidence is strongest at the pathway level; direct tests of NLRC3, NLRC5, and NLRX1 in acute infection, latency, and reactivation remain necessary. The evidence is asymmetric: NLRC3 has been functionally tested in HSV-1-infected cells and mice, whereas direct HSV-1 regulation by NLRC5 or NLRX1 remains unestablished. Comparative herpesvirus and pathway studies, therefore, support testable hypotheses for NLRC5 and NLRX1 in acute infection, latency and reactivation.

## 1. Introduction

Herpesviruses are large double-stranded DNA viruses that persist through lytic replication, latency and periodic reactivation. HSV-1 is a well-developed model for mucocutaneous, ocular and neurotropic herpesvirus disease [1]. HSV-2, varicella-zoster virus, human cytomegalovirus (HCMV), Epstein–Barr virus (EBV) and Kaposi’s sarcoma-associated herpesvirus (KSHV) provide complementary examples of immune evasion and latency. Host control depends on nucleic acid sensing, interferon induction, antigen presentation, mitochondrial stress control and cell-death regulation [2,3,4,5,6,7].

NLRs are intracellular immune regulators with shared nucleotide-binding and leucine-rich repeat architectures, but their functions extend beyond inflammasome formation. NLR biology now includes pathogen sensing, transcriptional control, interferon regulation, antigen presentation, mitochondrial signaling and metabolic adaptation [8,9,10,11]. This broader view matters in viral infection because viruses rarely perturb a single innate pathway; they reshape several host modules at once.

This review does not retell the canonical inflammasome-centered story. NLRP3, NLRP1, NLRP6, NLRP9, NLRP12 and NLRC4 remain important in viral inflammasome activation, IL-1β and IL-18 maturation, gasdermin-dependent pyroptosis and tissue inflammation [12,13,14]. Our focus is the regulatory NLR axis formed by NLRC3, NLRC5 and NLRX1. These molecules are better framed as modulators of cGAS-STING, MHC-I transcription and mitochondrial antiviral signaling than as classical inflammasome platforms [3,4,5,6,7,15].

Because direct HSV-1 evidence is established for NLRC3 but remains incomplete for NLRC5 and NLRX1, an HSV-1-centered synthesis should draw carefully on related herpesviruses. In this frame, HSV-1 remains the biological center, while HCMV (a betaherpesvirus), EBV and KSHV (gammaherpesviruses), along with related alphaherpesviruses, provide comparative evidence for conserved pressure on STING, antigen presentation and MAVS mitochondrial signaling [2,16,17,18,19].

Most NLR virus reviews still center on inflammasomes, pyroptosis or broad pattern recognition receptor signaling. We take a narrower route: regulatory NLRs beyond inflammasomes and their possible relevance to HSV-1 biology where direct HSV-1 data remain sparse. This framing produces a graded map of host-control checkpoints that can be tested experimentally.

## 2. Scope and Evidence Hierarchy

We distinguish four evidence levels. Direct HSV-1 evidence requires measurement or manipulation of the NLR in an HSV-1 infection model. Direct herpesvirus evidence applies the same criterion to another herpesvirus. Pathway evidence defines the relevant host mechanism without testing the NLR during herpesvirus infection, whereas hypothesis denotes an untested bridge to HSV-1 biology. NLRC3 reaches the direct HSV-1 level, although NLRC5 and NLRX1 do not. Many useful studies instead define pathway logic in non-herpesvirus infection, sterile inflammation, tumor immunology or non-human systems. We, therefore, grade the evidence rather than presenting every pathway link as an established herpesvirus mechanism.

We used narrative searches of PubMed and Google Scholar, cross-checked reference lists and DOI/PMID records, and included studies available through June 2026. Searches combined HSV-1 or herpes simplex virus with herpesvirus, NLRC3, NLRC5, NLRX1, cGAS-STING, MAVS, MHC-I or antigen presentation, latency, reactivation and immune evasion. We prioritized experimental studies that linked a virus or infection model to a host pathway, an NLR or a measurable readout. Broader reviews were used for mechanistic background or terminology.

Table 1 summarizes the evidence hierarchy used throughout this study. The classification is not intended to diminish pathway relevance; it prevents overclaiming. NLRC3 has strong relevance to DNA-sensing biology, NLRC5 to MHC-I transcription, and NLRX1 to mitochondrial antiviral signaling. For HSV-1, direct evidence supports NLRC3-mediated restraint of STING signaling, whereas the NLRC5 and NLRX1 models still rest mainly on antigen presentation and mitochondrial pathway evidence.

These labels are applied to each NLR rather than to the pathway as a whole. Direct HSV-1 evidence is available for NLRC3 from loss-of-function cell and mouse models [3]. Comparative herpesvirus evidence comes from other alpha-, beta- or gammaherpesviruses. Pathway evidence defines cGAS-STING, MHC-I, MAVS, ROS or autophagy biology without direct NLR testing in herpesvirus infection. A hypothesis marks links that remain untested in HSV-1.

## 3. NLRC3 at the DNA-Sensing Interface

NLRC3, also known as NOD3 or CLR16.2, is best positioned as a brake on excessive innate immune activation. It can restrain signaling downstream of the DNA sensor STING and reduce TBK1 and IRF3 activation after cytosolic DNA stimulation [3]. In cell-based studies, NLRC3 associates with STING and TBK1 [3]. It impedes the STING-TBK1 interaction and STING trafficking to perinuclear signaling compartments, limiting IRF3 phosphorylation and type I interferon output [3]. NLRC3 can also attenuate NF-κB signaling through TRAF6 [22]. The mechanistic basis for these interactions has been further contextualized in recent reviews on NLR regulation of type I interferon signaling [9,10]. Because these contacts occur at the STING-TBK1 node, the predicted HSV-1 phenotype is a shift in the threshold and duration of STING signaling rather than its complete loss. This mechanism is relevant to herpesvirus biology because viral DNA and infection-associated host DNA can trigger cGAS-STING surveillance during HSV-1 and other herpesvirus infections.

Beyond this direct HSV-1 evidence, comparative herpesvirus studies broaden the biological context. Porcine NLRC3 can bind short double-stranded DNA and regulate cGAS activation [23], and pseudorabies virus DNA has been reported to bind porcine NLRC3 and release NLRP3 inflammasome activation [20]. A gammaherpesvirus study also described bidirectional regulation between NLRC3 and latent gammaherpesvirus infection in B lymphocytes [21]. These comparative findings extend, rather than establish, the direct HSV-1-NLRC3 evidence and indicate that NLRC3 regulation may vary across herpesviruses.

Direct HSV-1 evidence shows that NLRC3 tunes STING-dependent responses. In HSV-1-infected Nlrc3-deficient mouse embryonic fibroblasts and macrophages, TBK1-IRF3 and inflammatory signaling increased; infected knockout mice showed higher early cytokine responses, lower HSV-1 genomic burden and reduced morbidity [3]. HSV-1 and related alphaherpesviruses encode cGAS-STING antagonists that suppress cGAS activation or degrade STING, while host factors such as TRIM23 couple cGAS to antiviral autophagy in anti-HSV defense [16,17,18,19]. Recent HSV-1 studies have also identified host factors that modulate cGAS- or STING-linked antiviral responses, including linker histone H1.2, LAPF-dependent lysosomal acidification and HDAC6-regulated STING/NLRP3 signaling [24,25,26]. The unresolved question is whether NLRC3 cooperates with viral antagonists to create a replication-permissive window or instead restrains immunopathology after antiviral defense has begun.

The functional interpretation of NLRC3 may also be species- and cell-context-dependent. Fish and mammalian systems show that NLRC3 can intersect with RIP2, STING, TBK1 and TRAF6-dependent inflammatory signaling [27]. HSV-1 studies should, therefore, avoid assuming one universal direction of effect. Knockout and rescue experiments need to measure viral genome copies, infectious titers, IFNB1 and ISG expression, cell death and tissue injury in the same system.

**Figure 1:**
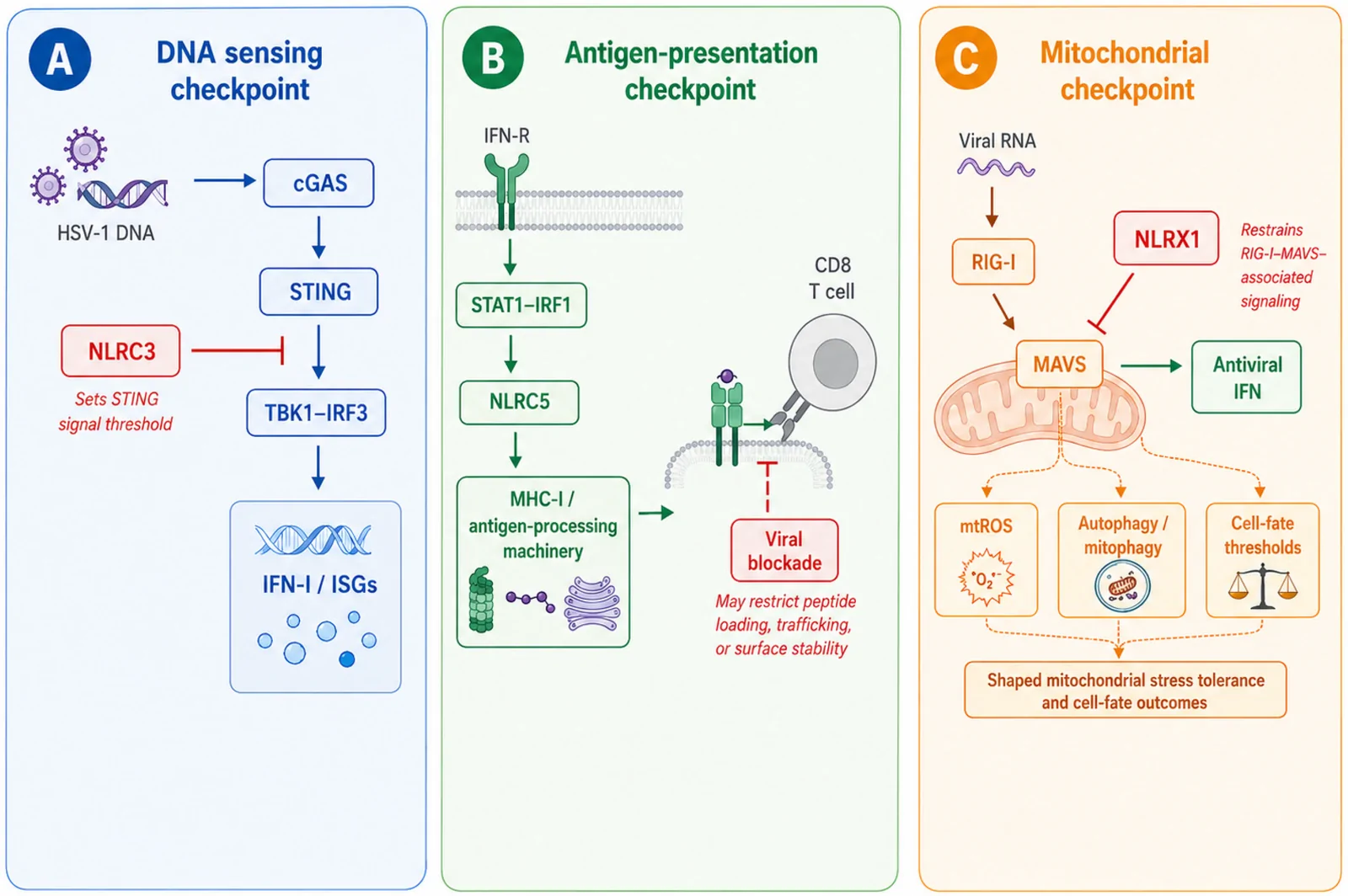
Three regulatory NLR modules proposed for HSV-1-centered herpesvirus infection. (**A**) NLRC3 is placed at viral DNA-cGAS-STING-TBK1-IRF3 signaling as a threshold-setting regulator. (**B**) NLRC5 links IFN/STAT1-IRF1 signals to MHC-I and antigen-processing machinery, whereas herpesvirus proteins may block peptide loading, trafficking or surface stability downstream. (**C**) NLRX1 connects RIG-I/MAVS-associated mitochondrial signaling with ROS, autophagy and cell-fate thresholds. The figure summarizes a pathway framework rather than proving that every link is directly demonstrated in HSV-1.

Thus, NLRC3-mediated restraint of STING-dependent antiviral signaling is directly supported in HSV-1 cell and mouse models [3]. What remains unresolved is whether a specific HSV-1 protein targets NLRC3, whether the effect is conserved in epithelial and neuronal models, and how signaling restraint influences tissue injury. Rescue experiments should, therefore, pair viral burden with STING-TBK1-IRF3 activation, IFNB1 and ISG expression and cell injury readouts.A schematic overview of the three regulatory NLR modules proposed in this framework is shown in Figure 1.

## 4. NLRC5 and the Antigen Presentation Layer

NLRC5 is the best-defined regulatory NLR in antigen presentation biology. It acts as an MHC class I transactivator by cooperating with RFX, NF-Y and CREB/ATF-family factors at SXY promoter modules. This complex drives transcription of classical MHC-I genes and components of the antigen-processing machinery [4,5]. NLRC5 also has context-dependent immune-regulatory effects, including enhanced TLR-induced NF-κB and type I interferon responses in NLRC5-deficient mice [28]. Its regulation involves nuclear import and export, interferon-responsive transcriptional activation and links with cellular metabolism [29,30,31,32]. NLRC5 itself does not bind DNA. It is recruited to the SXY promoter module as the specificity subunit of an MHC-I enhanceosome with RFX, NF-Y and CREB/ATF1. IFN-γ drives NLRC5 transcription through STAT1, creating a feed-forward loop that raises the surface MHC-I ceiling [4,5]. An HSV-1 protein that blunts STAT1 signaling would, therefore, be expected to lower this transcriptional ceiling, upstream of post-transcriptional viral blocks on peptide loading and trafficking. This logic parallels the STAT1-IRF1-NLRC5 targeting described for SARS-CoV-2 [33,34].

Herpesviruses are paradigmatic antigen presentation evaders. HSV-1 and HSV-2 can disrupt classical and non-classical antigen presentation, including MR1-dependent pathways [35,36]. EBV uses stage-specific strategies, such as BNLF2a-mediated TAP inhibition, BDLF3-mediated MHC-I downregulation, EBNA1 translation-linked avoidance and viral GPCR-dependent degradation of MHC-I molecules [37,38,39,40]. KSHV K3 and K5 downregulate MHC-I surface display through ubiquitin-dependent mechanisms [41,42], and HCMV modulates MHC-I at several replication phases while balancing NK cell recognition [43,44].

These multi-layered immune-evasion mechanisms across different herpesviruses and their interplay with the host NLRC5-MHC-I axis are summarized in Table 2.

These evasion strategies intersect with NLRC5 in two ways. If a virus blocks peptide loading, trafficking or surface stability downstream of transcription, increasing NLRC5 may not restore antigen presentation. If a virus suppresses interferon-STAT1-IRF1-NLRC5 signaling, NLRC5 becomes a more direct target layer. SARS-CoV-2 provides a useful non-herpesvirus example, in which the STAT1-IRF1-NLRC5 axis is targeted to reduce MHC-I induction [33,34]. For HSV-1, current evidence supports an indirect but important model: NLRC5 probably shapes the transcriptional ceiling for antigen presentation, while viral blockade may occur downstream.

Direct herpesvirus evidence is limited: NLRC5 has not been functionally manipulated in an HSV-1 infection model. The pathway evidence is nevertheless strong because NLRC5 controls inducible MHC-I transcription, whereas HSV-1 and other herpesviruses disrupt antigen presentation at several downstream steps [4,5,35,36,37,38,39,40,41,42,43,44]. The remaining inferential bridge is whether HSV-1 suppresses the STAT1-IRF1-NLRC5 transcriptional layer itself. Accordingly, NLRC5 clearly controls inducible MHC-I transcription, while herpesviruses often block antigen presentation downstream. HSV-1 experiments, therefore, need to ask whether the STAT1-IRF1-NLRC5 layer is suppressed, whether peptide loading or trafficking is dominant, or whether both occur in the same infected cell type.

## 5. NLRX1 as a Mitochondrial Checkpoint

Direct HSV-1 evidence for NLRX1 has not been established. NLRX1 is an atypical NLR linked to mitochondria and mitochondrial immune signaling. The original antiviral model placed NLRX1 as a regulator of mitochondrial antiviral immunity [6]. Later work connected NLRX1 with TUFM-containing complexes, type I interferon regulation, autophagy, ROS control and metabolic homeostasis [7,45]. The reported direction of NLRX1 activity varies across experimental systems. Studies emphasizing MAVS-dependent interferon often identify inhibitory effects, whereas studies centered on mitochondrial integrity, ROS, autophagy or tissue injury may identify protective host effects. These differences also reflect cell type, virus class, infection stage and endpoint selection; therefore, reduced cytokine production cannot be equated automatically with increased viral fitness or tissue damage. An N-terminal targeting sequence localizes NLRX1 to mitochondria. In the negative-regulatory model, NLRX1 associates with MAVS, interferes with RIG-I-MAVS assembly and engages autophagy through a TUFM-containing complex [6,7,45]. A parallel route in which NLRX1 promotes MAVS turnover has been mapped in hepatitis C virus infection via PCBP2 [46]. For HSV-1, this remains a cross-virus inference. Because NLRX1 operates near the MAVS node targeted by HSV-1 γ134.5 [47], viral restriction and host-injury readouts should be measured together rather than assumed to move in parallel.

This context dependence makes NLRX1 relevant to herpesvirus infection. HSV-1 triggers DNA sensing, RNA- or stress-sensing pathways, apoptosis, autophagy and mitochondrial remodeling. The HSV-1 accessory protein γ134.5 facilitates viral replication by disabling mitochondrial translocation of RIG-I [47]. KSHV can hijack PPM1G to restrict STING- and MAVS-mediated signaling [48], and HCMV UL37x1 can inhibit innate immunity and apoptosis to support efficient replication [49]. These findings do not prove direct NLRX1 involvement, but they place NLRX1 near mitochondrial signaling nodes targeted by herpesviruses.

The most useful HSV-1 hypothesis is that NLRX1 affects the balance between antiviral signaling and cellular stress tolerance. It could shape early MAVS cross-talk, mitochondrial ROS, autophagy/mitophagy, apoptosis resistance and inflammasome priming. Direct herpesvirus-specific NLRX1 data remain limited, and recent reviews of NLRX1 in viral infection emphasize the need to separate antiviral signaling effects from tissue-protective effects [46,50]. HSV-1 experiments should, therefore, measure both viral restriction and host-cell injury.

Thus, NLRX1 remains an HSV-1 hypothesis supported by mitochondrial pathway biology and indirect herpesvirus evidence, not a demonstrated HSV-1 mechanism. HSV-1 studies should separate NLRX1-specific effects from generic mitochondrial stress by pairing NLRX1 loss or rescue with MAVS localization, mitochondrial ROS, mitophagy or autophagy flux, cell death, viral genome copies and infectious titers in matched epithelial, myeloid and neuronal models.

## 6. HSV-1-Centered Model Across Infection Phases

HSV-1 provides a coherent center for this review because it engages all three regulatory layers. During acute epithelial infection, cGAS-STING, TLR and RIG-I-related pathways induce interferon and inflammatory cytokines [2]. During neuroinvasion and latency, viral gene expression is restricted. Immune pressure becomes compartmentalized, and neuron-intrinsic antiviral defenses become central. During reactivation, viral replication and inflammatory tissue injury both contribute to disease.

**Figure 2:**
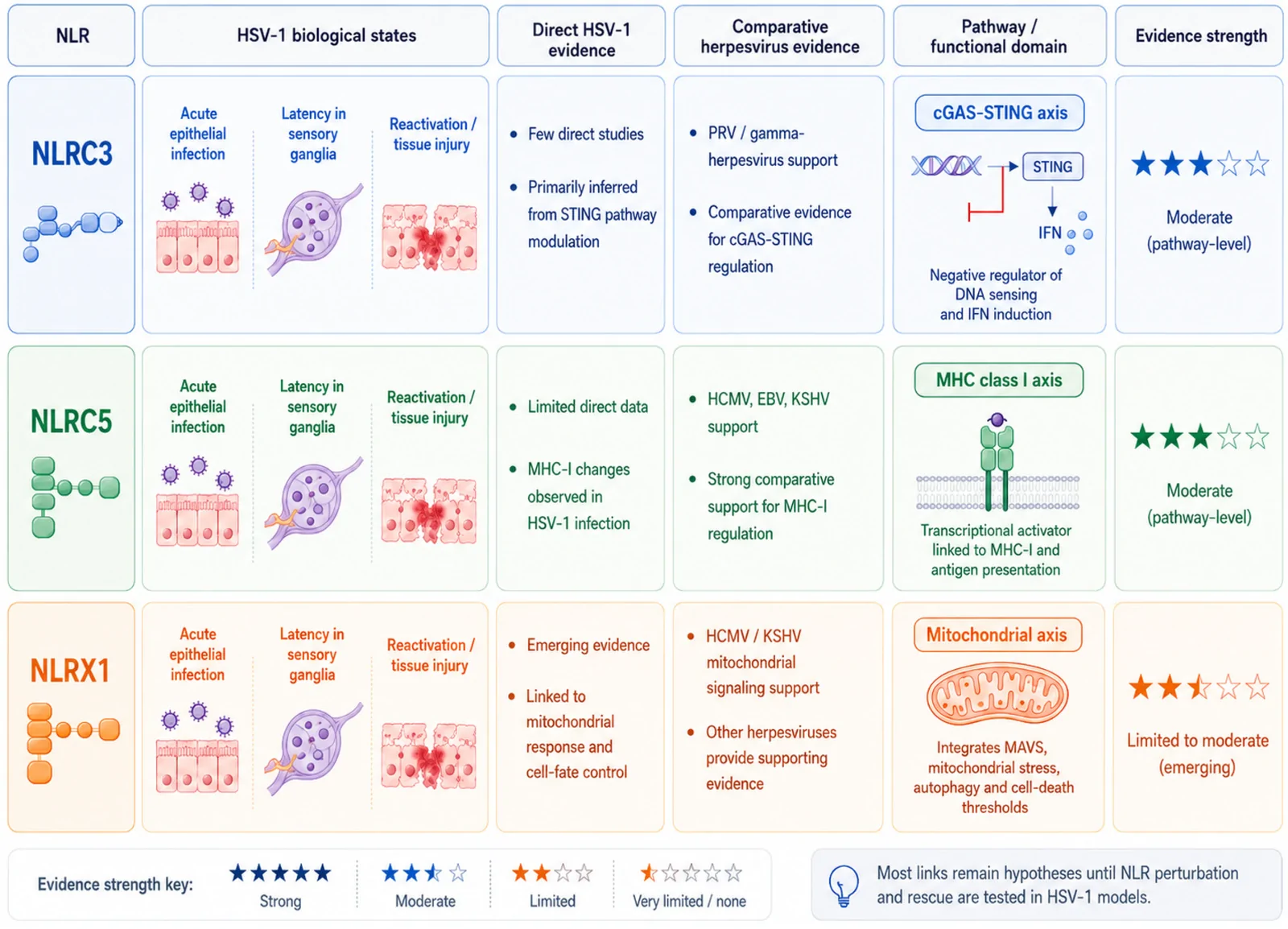
Evidence map for HSV-1-centered regulatory NLRs and comparative herpesvirus support. This schematic summarizes proposed placement of NLRC3, NLRC5 and NLRX1 across acute epithelial infection, neuroinvasion/latency and reactivation phases of HSV-1 infection. Direct HSV-1 cell and mouse evidence supports NLRC3-mediated restraint of STING signaling [3]. NLRC5 is supported by MHC-I transcriptional biology and herpesvirus antigen-presentation studies [4,5,35,36,37,38,39,40,41,42,43,44], whereas NLRX1 is supported mainly by mitochondrial-pathway and indirect herpesvirus evidence [6,7,46,47,48,49,50]. Comparative herpesvirus evidence provides guiding clues but cannot replace HSV-1-specific experimental validation.

The proposed model maps NLRC3, NLRC5 and NLRX1 onto this infection cycle as regulatory modules rather than isolated restriction factors (Figure 2). NLRC3 may tune early STING-mediated interferon amplitude. NLRC5 may influence how infected epithelial cells, keratinocytes, dendritic cells or macrophages communicate with CD8 T cells. NLRX1 may define mitochondrial stress tolerance, autophagy and cell-death thresholds in epithelial, myeloid and neuronal systems. At present, this model is stronger as a hypothesis-generating framework than as a completed HSV-1 mechanism.

Latency is the least established but most important knowledge gap. Evidence from T-cell immunity in HSV-1- and VZV-infected neural ganglia indicates that local immune surveillance and antigen presentation are central to persistent infection control [51]. However, there is not yet enough evidence to claim that NLRC3, NLRC5 or NLRX1 directly determines HSV-1 latency or reactivation. The right next step is cell-type-resolved measurement of these NLRs in trigeminal ganglia across acute infection, latency and reactivation, paired with viral load, interferon, MHC-I and mitochondrial readouts.

Accordingly, latency and reactivation are best framed as future-direction domains rather than established NLR mechanisms. The key value of the model is to specify what should be measured in trigeminal ganglia or neuron-enriched systems, not to claim that NLRC3, NLRC5 or NLRX1 already determine HSV-1 latency.

## 7. Comparative Herpesvirus Lessons

A comparative herpesvirus frame is useful because the same host pathways recur across the family. Alphaherpesviruses repeatedly target cGAS-STING, RIG-I/MAVS and autophagy pathways; HCMV provides a developed model of antigen presentation evasion; and EBV and KSHV show how latent and lytic programs reshape immune recognition, STING signaling and MHC-I display. KSHV miRNAs can target STING during lytic reactivation [52], EBV tegument protein BRRF2 can suppress cGAS phase separation [53], and a herpesvirus virion protein can directly inhibit cGAS DNA sensing [54].

The comparison is useful only if it stays pathway driven. Recurrent viral pressure on DNA sensing, antigen presentation and mitochondrial signaling can guide HSV-1 experiments, but it cannot replace them. This distinction is especially important when moving from epithelial infection to neuronal latency.

These examples do not make herpesviruses interchangeable. HSV-1 latency in sensory neurons differs from EBV latency in B cells or KSHV persistence in endothelial and lymphoid contexts. The comparison should, therefore, remain pathway driven. If multiple herpesviruses repeatedly target STING, MHC-I and MAVS/mitochondrial signaling, NLRC3, NLRC5 and NLRX1 are rational host-side candidates. The comparison should not be used to transfer virus-specific mechanisms without experimental validation.

## 8. Boundary with Inflammasome-Centered NLR Biology

Inflammasome biology remains relevant to herpesvirus disease, but here it is a boundary condition rather than the main subject. HSV-1 can activate STING-linked NLRP3 inflammasome responses [12]. NLRP12 has also been connected to antiviral RIG-I activation and herpes simplex keratitis models [13,14]. These studies are valuable for understanding inflammatory pathology, but they belong to a different conceptual axis from the regulatory NLRC3-NLRC5-NLRX1 framework. Keeping this boundary clear reduces overlap with prior inflammasome-focused NLR reviews.

## 9. Experimental Roadmap and Translational Caution

The translational value of regulatory NLRs lies in host pathway stratification, not immediate therapeutic claims. NLRC3, NLRC5 and NLRX1 may help identify whether a herpesvirus phenotype is dominated by excessive DNA sensing, poor antigen presentation or mitochondrial stress injury. Such stratification could guide model selection, biomarker design and rescue experiments, but it does not yet justify proposing NLRC3, NLRC5 or NLRX1 as clinical targets in HSV-1 disease.

Several experiments should take priority. First, measure NLRC3, NLRC5 and NLRX1 expression after HSV-1 infection in epithelial cells, macrophages, dendritic cells, sensory neurons and ganglion-derived models. Second, use knockout and rescue systems to test viral replication, interferon, MHC-I, ROS, autophagy and cell-death readouts in parallel. Third, ask whether known HSV-1 immune evasion proteins alter these NLRs directly or converge on the same downstream pathways. Finally, compare HSV-1 with HCMV, EBV and KSHV to separate conserved herpesvirus pressure from virus-specific mechanisms.

A ranked list of falsifiable experimental predictions, decisive assays and interpretive criteria for future HSV-1 studies is provided in Table 3.

The roadmap is a matrix of NLR perturbation or rescue, infected cell type and infection phase. Core readouts should be measured together rather than one at a time. These include viral genome copies, infectious titers, interferon and ISG induction, surface MHC-I or antigen presentation, mitochondrial ROS, autophagy flux, cell death and tissue injury markers.

## 10. Conclusions

This review places NLRC3, NLRC5 and NLRX1 in three herpesvirus-relevant host-control layers: DNA sensing, antigen presentation and mitochondrial antiviral stress. The evidence is unequal: direct HSV-1 cell and mouse studies support NLRC3-mediated restraint of STING signaling [3], whereas NLRC5 and NLRX1 remain supported mainly by pathway and comparative virus evidence. The framework is, therefore, practical; it defines where HSV-1 experiments should test NLR regulation, rescue and infection-stage specificity, while keeping comparative herpesvirus evidence in its proper role as a guide to experiments, not a substitute for them.

## Figures and Tables

**Table 1:** Functional positioning and evidence level of regulatory NLRs relevant to herpesvirus infection.

NLR	Dominant Host-Control Layer	Key Pathway Nodes	Herpesvirus Relevance	Evidence Level
NLRC3	Negative regulation of DNA-triggered interferon and inflammatory signaling	cGAS/STING, TBK1, IRF3, TRAF6, NF-κB	HSV-1 cell and mouse studies show that NLRC3 restrains STING-dependent antiviral signaling [3]; comparative herpesvirus studies extend this context [20,21].	Direct HSV-1 evidence in cell and mouse models [3]; comparative herpesvirus and pathway evidence [20,21]
NLRC5	MHC-I transactivation and context-dependent innate signaling	STAT1/IRF1, RFX/NF-Y/CREB, HLA-A/B/C, antigen-processing genes	Herpesviruses repeatedly interfere with antigen presentation; NLRC5 defines the host transcriptional capacity for MHC-I recovery [4,5].	Strong host MHC-I biology; comparative herpesvirus evidence; direct HSV-1-NLRC5 regulation remains limited
NLRX1	Mitochondrial antiviral signaling, ROS, autophagy and metabolic restraint	MAVS, TUFM, mitochondrial ROS, autophagy/mitophagy, NF-κB	Herpesvirus infection alters MAVS localization, mitochondrial integrity and autophagy, making NLRX1 a plausible checkpoint [6,7].	Mitochondrial pathway evidence; direct herpesvirus/NLRX1 evidence remains sparse

**Table 2:** Herpesvirus immune evasion mechanisms that intersect with the NLRC5-MHC-I axis.

Virus	Representative Evasion Layer	Relationship to NLRC5	Interpretive Caution
HSV-1/HSV-2	Disruption of classical and non-classical antigen presentation, including MR1 pathway impairment [35,36].	NLRC5 may set inducible MHC-I transcription [4,5], but post-transcriptional viral blockade may remain dominant [35,36].	Direct HSV-1 regulation of NLRC5 has not been sufficiently established.
HCMV	MHC-I downregulation during viral replication and signal-peptide-based immune evasion [43,44].	A comparator for transcriptional versus post-transcriptional antigen presentation control [43,44].	HCMV evasion is multilayered; NLRC5 is only one host-side layer.
EBV	BNLF2a, BDLF3, EBNA1 and viral GPCR mechanisms reduce antigen presentation at multiple stages [37,38,39,40].	Highlights latent and lytic pressures on MHC-I display [37,38,39,40].	B-cell latency biology should not be generalized directly to HSV-1 neuronal latency.
KSHV	K3/K5-mediated MHC-I downregulation and broader immune-evasion programs [41,42].	Shows strong gammaherpesvirus selection pressure on MHC-I surface display [41,42].	Mostly informs pathway logic rather than direct NLRC5-KSHV interaction.

**Table 3:** Ranked, falsifiable predictions for each NLR. Predictions are graded from P1 (strongest evidentiary basis) to P3 (most exploratory). For each prediction, we specify the decisive test, core readouts, and criteria for support or falsification.

Priority	NLR/Layer	Falsifiable Prediction (HSV-1)	Decisive Test and Core Readouts	Supported vs. Falsified
P1	NLRC5/antigen presentation	The STAT1-IRF1-NLRC5 axis sets the inducible MHC-I ceiling in HSV-1-infected cells, separate from downstream viral peptide-loading blocks.	NLRC5 knockout/knockdown and rescue in IFN-γ-treated, HSV-1-infected keratinocytes or epithelial cells; read out MHC-I and antigen-processing-gene transcripts, surface MHC-I and CD8 T-cell activation.	Supported if NLRC5 loss lowers inducible MHC-I and CD8 activation even when viral evasion genes are deleted; falsified if surface MHC-I is unchanged because post-transcriptional blockade dominates.
P1	NLRC3/DNA sensing	NLRC3 restrains STING-dependent interferon during acute HSV-1, widening a replication-permissive window.	NLRC3 knockout versus wild-type epithelial cells and macrophages with HSV-1, plus rescue; measure STING-TBK1-IRF3 activation, IFNB1/ISGs, viral genome copies and titers.	Supported if NLRC3 loss raises IFN-β/ISGs and lowers viral titers; falsified if interferon and replication are unchanged or move in the opposite direction.
P2	NLRX1/mitochondrial	NLRX1 sets the mitochondrial-stress and cell-death threshold in HSV-1 infection, separable from generic mitochondrial stress.	NLRX1 knockout and rescue in matched epithelial and neuronal HSV-1 models; measure MAVS localization, mitochondrial ROS, mitophagy/autophagy flux, cell death and viral titers.	Supported if NLRX1 loss shifts ROS, cell death and interferon tone beyond generic-stress controls; falsified if effects are indistinguishable from non-specific mitochondrial stress.
P2	Convergence (all three)	Known HSV-1 immune-evasion proteins act on shared pathway nodes (STING, STAT1, MAVS) rather than binding NLRC3, NLRC5 or NLRX1 directly.	Co-immunoprecipitation or proximity labeling of each NLR with candidate viral proteins; test whether NLR phenotypes persist after the pathway node is deleted.	Supported if no direct NLR-viral binding is found and phenotypes collapse when the node is removed; falsified if a viral protein binds and regulates an NLR directly.
P3	Latency/reactivation (all three)	Cell-type-resolved NLRC3, NLRC5 and NLRX1 levels change across acute, latent and reactivating infection in sensory ganglia and track local immune control.	Cell-type-resolved (e.g., single-cell) measurement in trigeminal ganglia across phases, correlated with viral load, interferon, MHC-I and mitochondrial readouts.	Supported if phase-specific NLR patterns correlate with control of reactivation; falsified if NLR levels show no relationship to infection phase.
P3	Comparative (all three)	Recurrent herpesvirus pressure on STING, MHC-I and MAVS predicts which NLR node is rate-limiting; HSV-1 shares the NLRC5 antigen presentation dependency with HCMV, EBV and KSHV through distinct upstream blocks.	Parallel NLR perturbation across HSV-1, HCMV, EBV and KSHV in matched cell types.	Supported if a shared NLR node with virus-specific upstream mechanisms emerges; falsified if no conserved NLR dependency is found.

## Data Availability

The data presented in this study are available on request from the corresponding author.

## Abbreviations

Abbreviations used in this manuscript.

Abbreviation	Definition
APM	Antigen-processing machinery
cGAS	Cyclic GMP-AMP synthase
EBV	Epstein–Barr virus
HCMV	Human cytomegalovirus
HSV-1	Herpes simplex virus type 1
IFN	Interferon
IRF3	Interferon regulatory factor 3
KSHV	Kaposi’s sarcoma-associated herpesvirus
MAVS	Mitochondrial antiviral-signaling protein
MHC-I	Major histocompatibility complex class I
NLR	Nucleotide-binding oligomerization domain-like receptor
ROS	Reactive oxygen species
STING	Stimulator of interferon genes
TBK1	TANK-binding kinase 1
